# Physicians’, Nurses’ and Pharmacists’ Perceptions of Determinants to Deprescribing in Nursing Homes Considering Three Levels of Action: A Qualitative Study

**DOI:** 10.3390/pharmacy8010017

**Published:** 2020-02-07

**Authors:** Rose-Anna Foley, Lucie Lechevalier Hurard, Damien Cateau, Daria Koutaissoff, Olivier Bugnon, Anne Niquille

**Affiliations:** 1 Center for Primary Care and Public Health (Unisanté), University of Lausanne, 1011 Lausanne, Switzerland; rose-anna.foley@hesav.ch (R.-A.F.);; 2 School of Health Sciences (HESAV), University of Applied sciences and Arts, Western Switzerland (HES-SO), 1011 Lausanne, Switzerland; 3 Institute of Pharmaceutical Sciences of Western Switzerland, University of Geneva, University of Lausanne, 1211 Geneva, Switzerland

**Keywords:** deprescribing, medication, nursing homes, elderly, nurses, pharmacists, physicians, interprofessional collaboration, qualitative study, caregivers

## Abstract

**Background:** Polypharmacy and the use of potentially inappropriate medications are frequent safety issues among nursing home (NH) residents. Deprescribing can significantly reduce the number of drugs used, medication costs, and mortality. This qualitative study sought to understand and compare the perceptions and practices of nurses, pharmacists, and physicians regarding deprescribing in Swiss NHs, referring to an implementation approach on three levels of action: the individual, the institution, and the healthcare system. **Methods:** Two focus groups were held with 21 participants: one focus group with 11 pharmacists, another with 10 nurses and six semi-structured interviews with physicians were conducted and focused on their individual experience and practices. They were audiotaped and fully transcribed, and a content analysis was performed using to MAXQDA (Ver 12) software. **Results:** (1) At an individual level, physicians were concerned by consequences of deprescribing in terms of safety. Nurses were closest to residents and stressed the importance of finding the right time, creating a bond of trust before deprescribing and considering the purpose of the stay in the NH. Pharmacists relied on structured guides for deprescribing, which led their reflection and practice. All professionals saw the complexity of the clinical situations, as well as residents’ and relatives’ fears of interruption of care. (2) At an institutional level, the professionals stressed the lack of time to discuss patients’ health and treatment, while pre-existing interprofessional collaboration, specifically, quality circles, seemed useful tools to create common knowledge. In order to reduce prescriptions, better coordination between physicians, nurses, pharmacists and specialists seemed crucial. (3) At the health system level, funding still needs to be provided to consolidate the process, go beyond organisational constraints and ensure deprescribing serves the patient’s wellbeing above all. **Conclusions:** At the individual level of implementation, the different healthcare professionals expressed specific concerns about deprescribing, depending on their defined role in NHs. Their perspective about the different levers to promote deprescribing at institutional and healthcare system levels converge towards interprofessional collaboration supported by the healthcare system. Specific funding and incentives are therefore needed to support a sustainable interprofessional team.

## 1. Introduction

Polypharmacy, commonly defined as the use of five or more concomitant drugs, and the use of potentially inappropriate medications (PIMs) are frequent problems among nursing home (NH) residents. Depending on estimations and definitions, the prevalence of polypharmacy ranges from 24% to 50% [1,2,3], and PIMs are prescribed to between 43% [4] and 90% [5] of NH residents. While polypharmacy is sometimes necessary to treat severe illness, and the use of a potentially inappropriate drug can be the best choice available for a specific patient, both are independently associated with worse health outcomes, lower quality of life, and increased risk of hospitalisation and death [1,6,7,8,9].

In this context, deprescribing, defined as “the process of withdrawal of an inappropriate medication, supervised by a healthcare professional with the goal of managing polypharmacy and improving outcomes” [10], seems a useful approach. Recent studies show that it can significantly reduce the number of drugs used by NH residents [11] and could reduce mortality [12] medication costs [13].

In Switzerland, no major initiatives to specifically promote deprescribing in NHs have been launched so far, either by health authorities or by professional societies. An explanation for this could be that, especially in NHs, deprescribing remains a complex matter, involving the active participation of residents, families, physicians, nurses and pharmacists, each group with its own goals and set of priorities [14]. Enacting these complex interventions depends on many determinants [15], such as the willingness to collaborate among health professionals or the organisation and financing of the healthcare system.

Supported by the Swiss National Research Programme 74 “Smarter healthcare”, our group launched a research project to study the effects and implementation of deprescribing interventions in NHs where interprofessional collaborations are already well-established [16,17,18]. To better design these interventions, understanding each groups’ needs and priorities is necessary. This qualitative study thus seeks to understand and compare the perspectives, needs and practices of health professionals active in Swiss NHs regarding deprescribing, and to conceive levers of action for implementing deprescribing in institutional care settings. To do so, the analysis will be focused on the determinants of deprescribing at three levels: the individuals’ concerns (health professionals, residents and relatives), the institutional issues and the healthcare system organisation as a whole.

## 2. Materials and Methods 

We conducted a qualitative study considering the meaning individuals give to health practices, in light of the contextual aspects underlying and influencing practices and discourses. Since this study aimed at informing a further intervention in NHs, we referred to an “applied” implementation science approach, considering that the determinants of implementation stand on multiple organisational levels, from the individual to the healthcare system as a whole [19], enabling to find effective levers to support the use of deprescribing in NHs, with a complex and critical view on the matter.

*Context of the study*: In the French-speaking cantons of Vaud and Fribourg, integrated pharmacist services (IPSs) are active and mandatory in all NHs, and are financed either by state funds (Vaud) or by the mandatory health-insurance scheme of Switzerland (Fribourg). These IPSs are based on interprofessional collaboration between pharmacists, physicians, and nurses, with the goal of optimising drug use in the NH. They consist of a yearly analysis, by its pharmacist, of each NH’s medication consumption regarding good prescribing practices and drug safety management in relation to up-to-date clinical and pharmaceutical evidence. This review is then discussed during one or more interprofessional quality-circle-type meetings throughout the year, with the goal of establishing a local treatment consensus [20]. Nurses and physicians then implement the agreed-on consensus, and its effects on drug use are assessed the following years. In these two cantons, all nurses and physicians are in regular contact with NH residents, whereas most pharmacists only supply drugs to the NHs and offer professional support on the good use of drugs.

The IPSs have been shown to reduce drug costs in NHs [18] and decrease the use of antibiotics [17]. Furthermore, they have fostered a climate of good collaboration between the professionals involved, which provided a good basis for the trial of deprescribing interventions. The NHs of these two cantons have thus been chosen for the larger research project on deprescribing intervention, which this study is a part of.

*Participant selection:* The focus groups were organised according to professional groups so that participants would feel free of any judgment induced by the implicit hierarchy between professions frequent in such settings, especially between nurses and physicians [21]. Participants were recruited between February and June 2017 following a purposive sampling approach aiming to account for the differences between cantons in the organisation of drug supply (hospital-type model in Fribourg, outpatient model in Vaud) and for the differences in points of view owed to different hierarchical positions, as well as to balance gender representation. Taking into account these three parameters led to rather large participation in the focus groups (see Table 1 for socio-professional components of focus group participants and interviewees). Two focus groups were held with 21 participants, the first with 11 pharmacists collaborating with NHs (7 men, 6 women), the second with 10 nurses working in NHs (7 women, 3 men). A third focus group gathering physicians active in NHs was cancelled due to insufficient recruitment. As an alternative, six physicians (3 women, 3 men) participated in semi-structured interviews, four led face-to-face and two over the phone.

*Data collection:* A semi-structured topic guide was developed with the goal of identifying barriers to and facilitators of deprescribing. A.N. (PhD, female) and D.C. (MSc, male), both pharmacists with experience in the NH setting and in quantitative research, first developed the topic guide based on their knowledge of clinical matters and of the NH setting. Qualitative researchers with extensive experience in social sciences in the health field and playing an active role in a qualitative research consulting team (D.K., MSc; R.A.F., PhD, both female) then reviewed and edited the final topic guide. Questions were adapted to the specificities of each professional group.

The pharmacists in the team (A.N., D.C.), who first contacted participants by phone or email, knew some of them from their previous or current practice. They provided participants with general information about the study and introduced the researchers conducting focus groups and interviews as members of the qualitative research platform. D.K. and R.A.F. conducted the focus groups and interviews without knowing the participants prior to the study; they asked participants open-ended questions about their practice in NHs and their points of view about medication and deprescribing. All interviews (duration 45–60 min, held at the physician’s office or by phone) and focus groups (duration 120 min, held in neutral conference rooms) were audio recorded and fully transcribed, and took place without the presence of other persons apart from the qualitative researchers mentioned above. No field notes were taken, apart from the order of speech of participants in each focus group.

Following the cancellation of the physicians’ focus group, the same topic guide was used for interviews, as we wished to compare the material collected from the three professional groups.

Due to recruitment impediments and for funding reasons, data saturation was not reached. Transcripts were not returned to participants, and their feedback was not sought on the findings. 

*Analysis:* The analysis focused on the differences between three professional statuses: pharmacists (Pha), physicians (Phy) and nurses (N). Their discourses were mapped to three concepts drawn from implementation sciences which structured our analysis, depending on whether discourses concerned what health professionals perceived and experienced (1) individually in the face of the clinical realities of the complex elderly patients they take care of, (2) the nature of their work in the NH or at a more macro-social level or (3) the organisation between these various professionals and their remuneration in their respective systems. A preliminary content analysis was conducted by discussing, comparing and identifying emerging themes in an ongoing process during data collection [22]. Once all interviews were complete, a computer-assisted analysis was carried out using MAXQDA (version 12, VERBI GmbH, Berlin, Germany). Interviews were coded separately by one researcher (L.L.H.) and crosschecked by another (R.A.F.). Themes were derived from the recorded material. The initial analysis plan called for the classification of identified themes between barriers and facilitators of deprescribing throughout the three implementation levels chosen as an organising principle. However, during analysis, it emerged that most themes could be classified as both barriers and facilitators, depending on the professional group responding. It was therefore decided to group similar themes by determinants of deprescribing, a more neutral concept not implying a positive or negative relationship.

*Reporting:* Consolidated criteria for reporting qualitative research (COREQ) were checked [23].

## 3. Results

While pharmacists interviewed seemed willing to put deprescribing into practice, nurses and physicians were more cautious; they considered it without having integrated it into their own practice. Pharmacists’ opinions in focus groups were therefore directly related to deprescribing, whereas those of nurses and physicians were often included in a more general reflexion on their practice.

### 3.1. Individual Level Determinants: Clinical Uncertainty and Psychosocial Aspects

#### 3.1.1. Numerous Medications Needed for Elderly Polymorbid Residents, Request for Specific Guidelines and Importance of Identifying Therapeutic Projects

According to professionals interviewed in this study, the numerous prescriptions received by NH residents corresponded first and foremost to their complex clinical reality:

“[…] these are polypathologies. If you have kidney failure, heart failure, plus Parkinson’s disease and diabetes, it will be very, very difficult to solve this with two or three medications.”

(Phy6M: the gender of the participant is indicated after each quote (M for male and F for female) to inform about gender representation.)

According to prompted physicians, numerous prescriptions are in most cases necessary because of many residents’ polymorbid profiles. Specifically, the high prevalence of dementia and psychiatric decompensations among the population they care for, combined with the current demographical situation of high age, was identified as leading to more polypharmacy.

Physicians felt that established treatment guidelines are ill-suited to their practice among polymorbid elderly persons living in NHs and contributed to detrimental polypharmacy instead of reducing it:

“[…] in the end if you look at the guidelines in each medical domain, our patient will get ten medications, because in fact he has diabetes, he has hypertension: he has polymorbidity.”

(Phy4F)

Physicians called for specific recommendations in NHs while pharmacists relied on structured guides for deprescribing, which scaffolded their reflexion:

“Where do I start […] I’m trying to get out of the woods and it is the old branch, this one, I can cut it off for sure, it is useless.”

(Pha10M)

For nurses, whose focus is more on the global situation of the person, the emphasis was on the importance of considering prescriptions in correlation with the purpose of the elderly person’s stay in the NH:

“What is the project? Going back home? To reenergise them? Or is it a palliative project and in this case we will concentrate on analgesic medication?”

(N2F)

The goals of care guided them in identifying which medicines are a priority and which, less important, can be removed.

#### 3.1.2. Fear of Destabilisation and Physicians’ Responsibility

In several instances, pharmacists described physicians’ concerns that prevent them from following a deprescribing approach: one considered that “*they are more timorous than we are”* (Pha5F), another said that “*physicians are extremely fearful of stopping a treatment”* (Pha7F) and a third recognised that this fear is sometimes founded and can be based on negative experiences with dose reductions, such as with proton-pump inhibitors (PPIs):

“They had trouble with people who had haemorrhages […] and who had to go back to the hospital. […] So they reintroduced them.”

(Pha11M)

A nurse mentioned her own fear of negative experiences while deprescribing:

“If a balance has been found, we’re not going to start taking something away and then struggle to find a new balance.”

(N8F)

Beyond the fear of destabilising the patient’s health, medication adherence was an important concern for physicians:

“I also try to avoid a breakfast made of medication […] rather, what matters is that the patient has his breakfast and is not nauseated and is compliant with the indispensable medications.”

(Phy1F)

This concern was even greater with patients who have difficulties swallowing their pills. This difficulty is perceived by nurses as an encouragement to limit the prescriptions:

“It makes us think quickly, to not prescribe too much, because one of the pills may not go down. So we need to choose the most important one.”

(N2F)

#### 3.1.3. Reluctance to Change to be due to Resident’s Age/Generation and Fears of Interruption of Care

All professionals perceived elderly residents as especially wary of, and unreceptive to, change in their medication because of their great age:

“It’s not easy for them to reconsider this. It’s not when they are 90 years old that they will reconsider things.”

(Pha4M)

A generational difference may also be involved: NH residents were seen as having an especially docile relationship to medicine: “*they have always obeyed to everything their physicians told them; they took everything they were told to take*” (N8F). This generation was thought to be used to a high medication consumption:

“The generation that is currently residing in homes is a generation used to treating each illness with a pill. We have a different level of awareness.”

(Pha5F)

Professionals often explained the difficulty in reducing medication by the resident’s and their relatives’ own difficulties in seeing that their situation has evolved towards an incurable or palliative condition:

“Some are hooked […] it may be because it is linked to the need to mourn their loss […] [Medication] represents the hope to be cured.”

(N8F)

Similarly, deprescribing may be perceived as withdrawal of treatment equating to the carer’s disinvestment.

*“For some residents, taking away some of their medication means we don’t really want to treat them, […] that death is near.”*

(Pha5F)

Residents’ resistance to change in their treatment was thought to be reinforced by their relatives’ attitudes. Professionals perceived families as actors who are often opposed to deprescribing. They would “put pressure” on maintaining the list of medication or even lengthen it, invoking the intimate knowledge they have of their parent’s situation and the destabilisation risk. A pharmacist stated:

“Some families play a significant role. The fact that they may have heard that the pink pill was removed is problematical to them.”

(Pha3M)

When residents suffer from cognitive disorders, relatives feel vested with a protective role, which may constitute a barrier to deprescribing:

“In psychogeriatrics, they get worried that their loved one is not able to defend himself, if we are in a context where the medication is not appropriate because he cannot say anything or understand what is at stake, they feel this responsibility to defend him and know on his behalf what medication he takes. So it is even more important to talk to caretakers when the patient has dementia.”

(Phy4F)

Some types of medication generate a strong attachment due to their action on particularly bothering symptoms linked to high age, such as constipation, sleeping disorders and anxiety. A nurse and a physician explained:

“The one that helps them sleep, they don’t forget (…) it’s also their purpose in life, to go to the toilet every day.”

(N7F)

“Everything that directly impacts their quality of life will be very difficult to touch.”

(Phy6M)

#### 3.1.4. Residents’ Self-Determination and Importance of Communication with Residents and Relatives

With a more critical perspective on how deprescribing may interfere with patients’ freedom to pursue medications despite their harmfulness, a pharmacist said:

“From a certain age, you obviously will have patients who will say, ‘I’m eighty years old, you’re not going to bother me about my sleeping pill, it’s the only thing that I have left, let me at least sleep well’ (…) it’s somewhat similar to the team who wants the patient to quit smoking when he’s 85 years old. In this case we are somehow thinking about end-of-life comfort, so to speak, I think we shouldn’t deprescribe just for the sake of it: ‘benzodiazepines are not good for you’, personally I feel it’s moralising.”

(Pha10M)

Patients’ self-determination may be in contradiction with a deprescribing project and cause it to be reconsidered. Therefore, in some cases, not deprescribing could be the most ethical practice if the patients wish to pursue some treatments that they consider important.

According to pharmacists, transparency of information is required on the potential harms and benefits of stopping a medication. According to them, good communication would be a facilitator to deprescribing:

“It may simply be a lack of communication. Because if we are patients, every day we receive a treatment and one morning, some things are missing because some people have decided for us, no one has explained anything to you, we don’t know why, this may be perceived as (...) pointless. One needs to understand this type of reaction. But if it is explained to the patient, it becomes obvious.”

(Pha6F)

#### 3.1.5. Finding the Right Timing, Rhythm and Conceiving Deprescribing as a Progressive and Reversible Process

According to pharmacists, admittance to the NH is conducive to a thorough review of treatments. Nurses pointed out the importance of acting at the right time and insisted on the importance of first creating a bond of trust with residents, knowing their personal history and getting to know their relatives:

“You have to get to know the patients and also their families and only then you declutter, you negotiate with the family to remove the medication that seems unnecessary.”

(N2F)

Therefore, a deprescribing process should not be set up until this bond has been created, usually a few weeks after the patient arrives. 

Physicians saw deprescribing as a long-term process and considered that negotiating with the patient must be done over a period of time in order to get results—over several months, for the following physician:

“I have in mind a patient who is ultra-polymedicated. […] As I got to see her psyche, and the fact that she was addicted to her medication, I told her […] we would have to reduce, but I gave myself […] one year for it.”

(Phy1F)

All participants agreed on the necessity of having a progressive deprescribing process in several steps: first a progressive dose reduction, followed by therapeutic windows, leading to withdrawal. For all professionals, an essential success factor of the process was its reversibility. Being able to go back if results are not satisfying is an important support in order to respond to the fear of destabilisation relayed by professionals, patients and family members. This reversibility may be facilitated by a transitory phase during which the removed drugs are kept “in reserve” and may easily be restarted.

*“I take it off but leave it in reserve. In one month, if we haven’t used it, we stop permanently. Reserves are a good tool to deprescribe, because it leaves some leniencies so that if things don’t go well, we can go back. It often works, and nurses know that they have tools in case it doesn’t work out. (…) It’s more there to reassure.”*

(Phy4F)

### 3.2. Institutional Determinants: Time Spent with Residents in NHs and Need for Interprofessional Collaboration

#### 3.2.1. Lack of Time with NH Residents

The lack of time available to discuss patients’ health and treatment with them was often mentioned to explain the difficulty in setting up a deprescribing plan. 

The pharmacist’s main role is to discuss prescriptions with professionals and not to interact with patients, as one pharmacist explained:

“We are not the ones interacting with residents; it is something that is totally beyond us. We are fine with explaining to the physician and the nurse, that we can do, but the resident’s individual problems are totally beyond our sphere of influence.”

(Pha5F)

The physician visits residents on a targeted, ad hoc basis. One physician talked about his difficulty to engage in a negotiation with one of the patients on the matter of deprescribing, considering the little time he has:

*“If I face resistance from a patient, when we know that from a medical standpoint, a medication doesn’t make much sense anymore, it’s often a question of time. Do I have time to talk for 30 min with them or do I postpone to the following month.”*

(Phy6M)

To compensate for staff shortages, an important risk was perceived in prescribing psychotropic drugs instead of nurses’ presence. A pharmacist says:

“Neuroleptics […] are sometimes there to compensate the staff shortage.”

(Pha1F)

One physician considered that if more time with challenging residents were available, especially during the night, it would be possible to replace psychotropic drugs with non-pharmacological approaches:

“I can imagine that if someone is slightly agitated one night, we may be tempted to give him what is in stock, may it be psychotropic or neuroleptic drugs, while all is needed is a glass of milk or a yogurt.”

(Phy1F)

#### 3.2.2. Autonomous Nurses Trained to Reduce Prescriptions and Favour non-Medicine Alternatives 

As physicians are only able to spend a short time within the NH, nurses often resort to suggesting a diagnosis and asking for a prescription over the phone. Some were satisfied with this level of autonomy.

*“I have a question, I get an answer; I’m fine with phone prescriptions. In any case, for us, with our attending physician, it is also a matter of trust.”*

(N7F)

Others felt that they are placed in a position of responsibility they should not have to assume and sometimes have to compensate for the physicians’ lack of presence:

“Personally, I find that rather nonsensical. Even though we have our own areas of expertise and we can describe the situation very well, it still seems to me that a prescription shouldn’t be done over the phone.”

(N1F)

Some physicians also perceived the nurses’ autonomy as a potential facilitator to adapt treatments to patients’ varying needs, thus reducing consumed drugs especially for reserve treatments. According to one physician, nurses unfortunately have a tendency to systematically ask for prescriptions.

*“It’s difficult for them to understand that they are the ones who decide when they should give it (…). That they can decide with the patient when it is necessary and when it isn’t. (…) Reserve medications remain in reserve! When I need it, I take it, when I no longer need it, I don’t take it anymore.”*

(Phy3M)

A head nurse highlighted that better trained nurses, who are more autonomous in their relationship with physicians, can contribute to limiting prescriptions.

*“If I have nurses who are slightly more confident, who will ask less of physicians, consequently I will have also fewer prescriptions coming back, and in the end we will use the non-pharmaceutical nursing set of skills.”*

(N2F)

#### 3.2.3. Referral to Specialists and Respect of Other Physicians’ Autonomy 

In addition to the lack of time for coordination between physicians and nurses on site, another barrier to deprescribing is added when specialist prescribing occurs. Professionals noted that general practitioners fear to suggest any changes to a specialist’s prescription and are even more reticent to contradict it, for instance when a resident comes back after a hospitalisation. This tension is particularly acute in psychogeriatrics:

“The problem is that it is the Word of God. When the psychiatrist speaks, (…) he is someone who is at the leading edge of knowledge in this particular field and (…) obviously the general practitioner (…) will not question his prescription.”

(Pha5F)

Some physicians find strategies to deprescribe “on the sly”:

“It’s really kind of taboo to go and say to a colleague, ‘don’t you think there are too many different psychotropic drugs, maybe you could stop one’. We don’t dare do that. It’s his patient; he’s the one who decides, and I don’t criticise that. […] Once I replaced him while he was on vacation and [a resident] is unwell and has four different types of medication, then I say (…) in the patient’s file: ‘The patient fell, he was confused, I stopped two psychotropic drugs’. I let my colleague know indirectly.”

(Phy2F)

#### 3.2.4. Interprofessional Communication Enabled by Integrated Pharmacist Services

All professionals highlighted the importance of smooth communication between them; the key to enhancing communication is, according to them, mutual knowledge and interpersonal relationships. 

IPSs, practised in all NHs included in the study, was often described as an efficient tool to create common knowledge useful for interprofessional collaboration and is a potential lever for deprescribing. 

Nurses highlighted the usefulness of having a pharmacist within their structure and the complementary benefit of mutual knowledge, which facilitates a reflective process on how medication is used:

“They are real discussions and we can see that they lead to good results. I can see real changes, it’s really good, it really improves medication practices.”

(N4F)

A pharmacist applauded the efficiency of this approach:

“We created quality circles, there has been a drastic reduction of prescription or dosage and I think that had we not come and discussed it with the physicians, the curve would have remained low. We played a very important role there; we weren’t just initiators.”

(Pha1F)

Engaging in an efficient deprescribing process is often the result of an individual initiative. However, the professionals who were interviewed mentioned that collaborative practices with regard to IPS must be established as part of the NH organisation to reduce the risks associated with polypharmacy including establishing pharmaceutical thresholds, regular monitoring and reassessment of long-term prescriptions, the transmission of pharmaceutical information, and the collaborative management of reserve medications. 

### 3.3. Healthcare System Determinants: Funding Time for Deprescribing and Need for Consistency within the Care Team

#### 3.3.1. Lack of Funding for Comprehensive Treatment Reviews

The funding available for the professionals’ activity plays a vital part in the implementation of a deprescribing process. One physician highlighted the lack of funding for comprehensive treatment reviews and its consequence when it comes to managing prescriptions on a “one-off” basis for each new symptom.

*“In a NH context, the whole polypharmacy thought process is performed when the patient is not there. Insurance companies don’t understand that you spend more time in the absence than in the presence of the patient. We’re up against a brick wall right now. At the same time, we can’t do a good job if we don’t know the diagnosis and if we don’t think about it carefully and if we don’t pick up the phone and call the pharmacists… the question is how this time can be paid.”*

(Phy1F)

#### 3.3.2. Remuneration of the Pharmacist Based on Medication Delivery versus Pharmaceutical Services

Pharmacist’s work is highly dependent on the funding of drugs supplied within the NH: in the canton of Vaud, the outpatient system pays pharmacists for each individual medication provided, thus incentivising medication sale, while in the canton of Fribourg, the lump sum system valorises more prescription optimisation.

All pharmacists interviewed noted to what extent the IPS in place enhances their expert roles within NH teams, including financially, considering that they receive specific payment for it. 

They highlight, however, the importance of being mindful of potential abuse of a system in which pharmaceutical competencies are geared towards deprescribing with financial objectives in mind, rather than the patient’s wellbeing. Pharmacists considered they have to both deprescribe and ensure that deprescribing happens in a fair and equitable manner, even facing patients who receive expensive treatments:

“If there really is a treatment that ought to be given, to my mind, it has to be given whether it is expensive or not. We have to be careful that there aren’t any treatment restrictions.”

(Pha2F)

One physician also noted a possible medical abandonment tendency:

“There is kind of trend of saying, ‘they are in a NH, they no longer need that’.”

(Phy1F)

Deprescribing consequently appeared to be associated with the sensitive matter of therapeutic limitations elderly people may face, as well as financial incentives for NH, depending on the health system organisation. 

#### 3.3.3. Multiplicity of Health Professionals versus Referents

Beyond the financial aspects, the health system also has an impact on interprofessional collaboration, depending on which actors will be authorised. The fact that each resident can choose to keep their own physician, rather than the attending physician of the NH, was described as a factor multiplying the number of actors involved in the care of patients, thus increasing the difficulty to reach a consistent medication policy. One nurse explained the difficulties of dealing with multiple physicians who only come occasionally, instead of one attending physician who would give common directives:

“We have to work with 10 different physicians who don’t have a common philosophy. Not all physicians will have the same attitude. They all come on their own time, (…) once they have seen all the patients, at six in the evening, and we are pretty busy at that time.”

(N9F)

An attending physician would also enable for regular links with outside partners, such as specialists or hospitals in the area, to be established.

## 4. Discussion

Based on identified determinants raised by each health professional group (summarised in Table 2), and which can be complementary, we discuss ways of action to support a deprescribing process at each of the three levels (individual, institution, health system). Nurses are those who spend most time with residents. They mostly identified practical and clinical issues, while above all being concerned with their role in order to maintain a good relationship with residents and establish a therapeutic project with them in which deprescribing can be integrated. Physicians are responsible for the prescription and were highly concerned about consequences of deprescribing in terms of safety and residents’ wellbeing. While not in contact with residents, pharmacists wished to communicate openly with the health professionals, as well as with residents, and had a specific idea of how to implement deprescribing in the institutions and support it at a health system level. 

### 4.1. Individual Level: Working on Pedagogy while Respecting Complex Patient-Centred Care

NH residents are usually complex patients with polymorbidity and consecutive polypharmacy issues, according to the health professionals who took part in this study. Clinical issues with deprescribing are well understood, but the psychosocial ones remain a challenge. The fears and concerns of residents and families about deprescribing identified by the professionals in this study echo the ones found in the literature, namely the fear of symptom reappearance [24,25] and the perception of deprescribing as giving up on residents’ care [25]. However, in another part of this study based on the residents’ points of view [26], results hinted toward residents’ willingness to take less medicines and the importance of searching ways to go beyond their fears through better communication, also suggested by other authors [27]. The importance of collaboration with patients and relatives has been stressed by healthcare professionals in other studies, though they noted that patients and families are sometimes insistent on continuing specific medications [28].

In this study, the health professionals identified multiple, complementary ways to address these concerns. Shared decision-making, a concept at the core of deprescribing, is the most prominent of them [29]. Adopting a more patient-centred approach, taking into consideration the personal life goals, individual struggles, such as swallowing difficulties, and literacy level of residents could help professionals define priority medicines in a complex drug regimen and adapt the level of pedagogy to deploy. Patient education about the potential benefits and harms of deprescribing is another way to address patient’s concerns; in that regard, the development of communication tools, such as those offered by the Canadian Deprescribing Network [30], seem necessary. Motivational approaches, eventually including the residents’ relatives, could also be leveraged.

Shared decision-making and education, however, must not be a façade for imposing professional’s views on the resident or be moralising, as pointed out by pharmacists. Indeed, the risk is that inherent pressure of the NH as a “total institution” [31] tends to disempower residents, especially with regard to their medication. Turner et al. (2016) [32] reported that it is important for residents to feel that they have the right to question their physician about their medication and the right to continue taking medications that they feel are good for them, even when it conflicts with clinical evidence. 

Finding the right time to initiate a deprescribing process and the right rhythm for it, sometimes over months, was reported mostly by nurses as a major way to increase patients’ acceptance. Collaborative approaches also seem necessary and include all involved professionals, from those who are in a day-to-day relationship with the residents and their relatives and who can identify difficulties residents have to swallow medicines, for instance, to those who seldom see the residents. In this regard, collaboration must be developed beyond the IPS actually in place, as it does not address individual patients’ situations, but focuses more on NH-wide medication optimisation consensus.

Each medical association engaged in geriatrics or any specialty treating chronic conditions should develop specific guidelines for elderly, polymorbid patients, as suggested by physicians. Pending these guidelines, health professionals can rely on the safety evidence from a small but growing number of deprescribing studies. Indeed, the literature describes only a few cases of necessity for reintroduction of withdrawn or reduced medicines [33]. The evidence of the literature confirms that the clinical skills and knowledge engaged for prescribing and deprescribing are similar. Field professionals have to be more aware of the evidence supporting deprescribing to be confident enough to start. Within the IPS in NHs, pharmacists can objectively and effectively communicate this evidence to physicians and nurses within the IPS. Besides that, the positive impact of deprescribing on clinical outcomes and quality of life is still debatable and studies are still needed [12]. However, the efforts that must be made to correct the negative consequences of over-medication must not obscure the fact that under-medication also frequently exists in the elderly. The analysis of an individual resident’s medication should also pay close attention to this phenomenon.

### 4.2. Institutional Level: Finding Time and Improving Interprofessional Collaboration

All professional caregivers agreed that a patient-centred approach is needed, but organisational constraints are a strong impediment to its realisation. Similar results have also been highlighted in a recent narrative literature review on deprescribing [10], and patient-centred interventions have been shown to be more effective than other types of interventions [12]. Time is required to develop meeting spaces with residents; integrate a regular medication regimen review in the routine operations of a NH; complement the work done by the IPS in NHs (i.e., interprofessional quality circles optimising therapeutic classes prescribing) with individual medication file reviews. 

Non-pharmacological approaches that reduce the use of certain medications while continuing to care also require additional professional time with residents, as raised by all three health professional groups in this study. Setting up activities, dedicating attendance time to the individualised accompaniment of a person who for example sleeps badly at night seems particularly effective. All this represents additional staff costs that the institution must be able to organise and finance. 

One of the particularities of NHs is the polymorbid population it accommodates, which requires the intervention of a plurality of actors (general practitioners, NH referring physicians, hospital specialists, pharmacists, nurses, etc.). Their collaboration is essential, yet differences in status, involvement in the institution and symbolic hierarchies between them can be obstacles to the optimisation of prescriptions. The particular institutional context of the NH also implies organising interprofessional collaboration in a different way from an ambulatory setting. In particular, the boundaries of professional jurisdictions, according to Abbott’s concept (1988) [34], need to be more flexible. Indeed, nurses, because of their daily proximity to residents, have valuable assets like capacity for observation, situation analysis and rapid adaptability. Primary care providers, including nurses, are often cited as key players, particularly when they are designated as referents for each resident. However, their autonomy of intervention is not always sufficiently emphasised or even accepted by themselves as pointed out by nurses and physicians. It is therefore important that the nurses feel supported in their capacity to suggest alternatives to medication, while maintaining consistency with the prescribed treatment, through a reflexive approach and frequent discussions with the physicians. Similarly, the fact raised by physicians that they do not feel comfortable adapting prescriptions made by colleagues, appears counterproductive. These territorial boundaries are the result of strong professional cultures, which are difficult to change as a whole; they seem not to be specific to Switzerland, as physicians in other settings reported the same hesitancy to address drugs prescribed by a colleague [35]. However, the development of collaborations in a network of stable external partners regularly mobilised by the NH team (that goes from the general practitioner to specialists and even to the surrounding hospitals) could contribute to optimise drug consumption. Literature shows that all healthcare professionals are capable of leading successful deprescribing interventions, which suggests that these could be adapted to fit a particular organisational culture without losing their impact [28].

In the end, it is useful to remember that time is often not the primary barrier: we lack time when we lack practice or legitimacy, when collaboration is not well established, when changing a habit, and when there is no specific remuneration. It therefore seems necessary to consider deprescribing as generating new practices that deserve their place in the NH. These possibly imply additional financial charges but can also provide benefits for the NH offsetting those costs, such as directly decreasing costs related to drug reduction and their negative clinical outcomes (risk of falling, emergency hospitalisation), as well as increasing resident autonomy and well-being.

### 4.3. Healthcare System: Coherent and Stable Healthcare Teams Paid for the Work Related to Deprescribing

At the level of the health system, which is cantonal, it appears from discourses of the representatives of the three health professions that the approach that promotes the stability and coherence of the teams of professionals working in the same NH is the most conducive to inter-knowledge and to the development of smooth communication. For example, the fact that a single physician is a referral for all residents of an NH constitutes a facilitator. Indeed, he/she represents a single and regular interlocutor for the care teams and residents and can collaborate in a coherent manner with the hospital partners, specialists and pharmacists. By extension, having a referent pharmacist, as it is the case with IPS in NHs, appears as an additional facilitator. 

To ensure this consistency and stability of teams, it is essential that the health system provides funding for the remuneration of the various tasks contributing to the implementation of a process. For physicians, this includes the remuneration of the analysis of files without the patient present, as well as attendance at interprofessional sessions. For pharmacists, the remuneration of their work for the IPS in NHs, which could be extended to analyses of individual medication records, is regularly cited as a facilitator of a deprescribing process. 

Healthcare professionals have reported the same concerns in other studies [35], stressing the importance of interprofessional teams for successful deprescribing, and the frequent time constraints imposed by under-staffing and the high administrative load.

### 4.4. Limitations and Perspectives

This study took place in two cantons where strong interprofessional collaboration already exists. It does not cover the views of professionals in cantons where no such collaboration exists. In addition, this study involved participants only from the French-speaking part of Switzerland and does not account for the important cultural differences with the German- and Italian-speaking parts of the country.

The point of view of physicians could be represented differently in this study compared to those of nurses and pharmacists, because of the different data collection method used. Focus groups tend to produce more varied discussions among the group, and less personal experience [21]. However, the authors estimate the risk of bias to be low, as the material collected from physicians showed to be similar in ways of expressing and themes identified to those collected with nurses and pharmacists. 

Finally, pharmacists are the only healthcare professionals represented within the authors of this paper; this may result in a better understanding of their concerns than those of other professionals.

Further studies in other cantons, particularly in the German-speaking part of Switzerland, are needed to assess the influence of local interprofessional relationships, healthcare organization, and policy on actively deprescribing inappropriate medications in NHs.

## 5. Conclusions

As physicians, nurses and pharmacists have different professional roles, they expressed specific but somehow complementary concerns about deprescribing at the individual level. Physicians are responsible for prescribing and were highly concerned by the consequences of deprescribing in terms of safety and patients’ wellbeing. Nurses, who spend the most time with residents, above all sought to maintain a good relationship with patients and to establish a therapeutic project with them, in which deprescribing can be integrated. While not in contact with residents, pharmacists had a specific idea of how to implement deprescribing in their institutions with a global health system view and wished to communicate openly with the other professionals as well as with patients. 

The perspectives of all professionals about the different levers to promote deprescribing at the institutional and healthcare system levels seem to be more consistent. An interprofessional approach seems essential to overcome the barriers identified at the individual level and to deprescribe efficiently in NHs with a resident-centred care perspective. In order to benefit from this synergy between the specific approaches and competencies of each professional group, the organisational structure of the work should be rethought to foster interprofessional collaboration. This could partly compensate the extra time needed, but specific funding should be provided to consolidate the process of deprescribing in NHs. Besides, the healthcare system must provide incentives to support sustainable interprofessional teams within the NHs.

## Figures and Tables

**Table 1:** Socio-professional components of focus group participants and interviewees.

Professional Status	Sex	Hierarchy Level	Work Location (FR/VD)
Pharmacists (n = 11)	5 men 6 women	4 owners 7 employees including 1 manager	Canton FR = 5 Canton VD = 6
Nurses (n = 10)	3 men 7 women	3 head nurses 7 standard nurses	Canton FR = 6 Canton VD = 4
Physicians (n = 6)	3 men 3 women	4 head physicians 2 general practitioners	Canton FR = 2 Canton VD = 4

FR: Fribourg; VD: Vaud.

**Table 2:** Determinants to deprescribing identified by nurses, pharmacists and physicians.

		Nurses	Pharmacists	Physicians
**1. Individual determinants**	**Clinical uncertainty facing complex NH residents**			
	Numerous medications needed for high age polymorbidity			**X**
	Guidelines for elderly polymorbid patients vs. by pathology		**X**	**X**
	Adaptation of the medication to the patient’s project	**X**		
	Fear of destabilisation and physician’s responsibility		**X**	
	Concern for adherence and elderly’s difficulty in swallowing	**X**		**X**
	**Psycho-social aspects**			
	Reluctance to change due to residents’ age/generation	**X**	**X**	**X**
	Deprescribing seen as interruption of care	X	**X**	**X**
	NH residents very attached to their medicines	**X**		**X**
	Residents’ self-determination		**X**	
	Importance of communicating with residents and relatives		**X**	
	Finding the right timing and rhythm for deprescribing	**X**		**X**
	Deprescribing as a progressive and reversible process			**X**
**2. Institutional determinants**	**Time spent with residents**			
	Lack of time with NHs residents		**X**	**X**
	Autonomous nurses trained to reduce prescriptions and favour non-medicine alternatives	**X**		**X**
	**Interprofessional collaborations**			
	Deference to specialists and respect of other physicians’ autonomy		**X**	**X**
	Interprofessional communication enabled by integrated pharmacist services	**X**	**X**	
**3. Healthcare system determinants**	**Funding time for deprescribing**			
	Lack of funding for comprehensive treatment reviews		**X**	**X**
	Remuneration of the pharmacist based on medication dispensation versus pharmaceutical services		**X**	
	**(In)consistency within the care team**			
	Multiplicity of professional caregivers versus referents	**X**		

NH: Nursing home.

## Data Availability

Most data generated or analysed during this study are included in this published article. The full datasets generated are available from the corresponding author on reasonable request.

## Abbreviations

List of abbreviations:

F	female
IPS	integrated pharmacist services
M	male
N	nurses
NH	nursing home
Pha	pharmacists
Phy	physicians
PIM	potentially inappropriate medication
PPI	proton-pump inhibitor
